# Neutrophil Extracellular Traps and Respiratory Disease

**DOI:** 10.3390/jcm13082390

**Published:** 2024-04-19

**Authors:** Paul T. King, Lovisa Dousha

**Affiliations:** 1 Monash Lung, Sleep, Allergy and Immunology, Monash Medical Centre, 246 Clayton Rd, Clayton, Melbourne, VIC 3168, Australia; lovisa.dousha@monash.edu; 2 Department of Medicine, Monash University, Clayton, Melbourne, VIC 3168, Australia

**Keywords:** neutrophils, leukocytes, extracellular traps, respiratory, inflammation, diagnosis, therapeutics

## Abstract

Extracellular traps made by neutrophils (NETs) and other leukocytes such as macrophages and eosinophils have a key role in the initial immune response to infection but are highly inflammatory and may contribute to tissue damage. They are particularly relevant to lung disease, with the pulmonary anatomy facilitating their ability to fully extend into the airways/alveolar space. There has been a rapid expansion in the number of published studies demonstrating their role in a variety of important respiratory diseases including chronic obstructive pulmonary disease, cystic fibrosis, bronchiectasis, asthma, pneumonia, COVID-19, rhinosinusitis, interstitial lung disease and lung cancer. The expression of NETs and other traps is a specific process, and diagnostic tests need to differentiate them from other inflammatory pathways/causes of cell death that are also characterised by the presence of extracellular DNA. The specific targeting of this pathway by relevant therapeutics may have significant clinical benefit; however, current clinical trials/evidence are at a very early stage. This review will provide a broad overview of the role of NETs and their possible treatment in respiratory disease.

## 1. Introduction

The respiratory tract is constantly exposed to a variety of microbes and inhaled substances, which may be pathogenic. Leukocytes have a variety of mechanisms to protect the host, one of which is the expression of extracellular traps; these have been best described as being produced by neutrophils (NETs), although other cell types such as macrophages (METs) and eosinophils (EETs) may also express them. The vast majority of the published literature describes NETs.

Extracellular traps such as NETs are typically expressed as a response to infection but may also occur in response to other inhaled stimuli. This induces a series of intracellular changes, resulting in the extracellular expression of DNA/chromatin with co-expressed mediators including protease and histones, which trap and kill microbial pathogens. NETs play an important role in host defence and are highly inflammatory. As such, they can potentially contribute to lung damage and be a “two-edged sword” [1,2,3]. In, addition extracellular traps also have some role in the resolution of inflammation by removing cellular debris [4,5].

Takei et al. described the induction of a specific pathway of extracellular DNA expression after stimulation with a mitogen [6]. Brinkmann et al. described how neutrophil extracellular traps are formed to kill bacteria and introduced the term of NETs, describing the colocalization of the chromatin with granule proteins [7]. Over the past 20 years, there has been an exponential increase in the publications in this area. The lung may be particularly relevant to the action of extracellular traps as its alveolar and bronchial space permits the full extension of the extracellular component to catch pathogens, in contrast to solid organs.

This review highlights the role of NETs and other extracellular traps in respiratory disease. It concentrates on the mechanisms involved in their expression, methods that can be used to detect their presence, potential roles in respiratory pathogenesis and how the inflammatory effect can be therapeutically targeted.

## 2. Lung Immunology

The immune response protects the lungs from the effects of both microbial pathogens and non-microbial substances; perhaps no other organ in the body is so exposed. Pathogens include bacteria, viruses and fungi. Most bacteria that cause respiratory disease are part of the upper respiratory microbiome that includes both long-term and short-term colonising microorganisms, and are opportunistic pathogens that generally cause disease when there is a defect in host defence [8,9]. In contrast, viruses are acquired externally and are frequently highly contagious with acute clinical manifestations. Lung fungal infections are generally opportunistic microorganisms widely distributed in the environment, that cause clinical disease in immunocompromised hosts [10]. There are many non-microbial substances that are responsible for lung disease and some of the most important include cigarette smoke, air pollution, air borne allergens such as house dust mites and pollen and industrial products such as silica dust.

The innate immune response, comprised of structural and cellular components, is the first line of defence in the lung. The structural defences of the respiratory system include cough, air movement and particularly the barrier function of the epithelium and the mucociliary apparatus [11]. The cellular defence is mediated by macrophages and neutrophils. Alveolar macrophages kill invading pathogens but also recruit other components of the immune response starting with neutrophils [12]. Neutrophils are recruited in large numbers to kill pathogens and are highly inflammatory. Subsequently, the adaptive immune responses are activated and are primarily driven by lymphocyte responses with the production of antibodies, cytokines, and cytotoxicity [13]. If the threat cannot be completely removed (e.g., bacterial colonisation), a persistent inflammatory state with clinical disease may result; in this circumstance, the host immune response may have a key role in the disease’s pathogenesis [14].

Macrophages and neutrophils are designated as being phagocytes whose chief functional role is to phagocytose and remove pathogens. However, the immune system is complex with significant redundancy and the formation of extracellular traps is important in host defence and is a rapidly evolving area.

## 3. Mechanisms of the Formation of Neutrophil Extracellular Traps

The mechanisms of extracellular trap formation have still not been fully defined. The immune system has significant redundancy with different pathways being employed to achieve outcomes, and this has been demonstrated to be the case in NET formation. The study of neutrophils is challenging due to their short life span, instability and lack of cell lines [3].

The primary function of NETs is to protect the host against infection, and a wide variety of microbial pathogens have been described that induce their expression including bacteria, fungi and viruses. NETs can attack pathogens that are too big to phagocytose such as fungi. In addition, non-microbial stimuli including cigarette smoke exposure and gout crystals can induce NET expression.

The most well-defined pathway of NET formation is driven by the formation of the oxidative burst and results in the death of the neutrophil (NETosis) [15], and this process is quite distinct from other pathways of cell death such as apoptosis and necrosis [16]. Stimulation (e.g., infection) activates the formation of the nicotinamide adenine dinucleotide phosphate (NADPH) complex and the formation of reactive oxygen species (ROSs) [17]. Neutrophil elastase (NE) and myeloperoxidase (MPO) are released from azurophilic granules [18]. NE and MPO cleave histones and peptidylarginine deiminase (PAD) 4 citrullinates histones (H3Cit) [19] which decondense chromatin. Pore forming proteins (e.g., gasdermin) permeabilise the plasma membrane [20]. This leads to the rupture of the cell membrane [16] and the expression of multiple strands of extracellular DNA with co-expressed proteases (e.g., NE) and H3Cit to form an NET to trap and kill pathogens, and results in the death of the neutrophil; it has been designated as suicidal NETosis.

**Figure 1:**
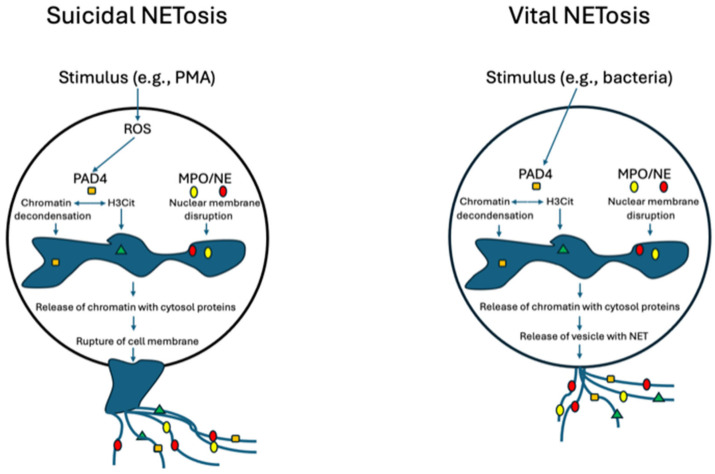
Neutrophil extracellular trap formation.

NET formation may also occur without the death of the cell, designated as “vital NETosis” [21,22]. This pathway may occur without the involvement of the oxidative burst and is triggered by an increase in intracellular calcium leading to expulsion of the chromatin and co-expressed granule proteins. As the plasma membrane remains intact, the neutrophil is still viable and retains its functional capacity (e.g., chemotaxis). This process occurs within minutes of neutrophil activation (e.g., by pathogen exposure). Figure 1 demonstrates suicidal and vital NETosis.

The instigating factors for suicidal and vital NETosis do appear to overlap to some extent particularly in infectious causes and the why some factors initiate one pathway over the other is not well understood. However, certain stimuli such as mitogen phorbol myristate acetate (PMA), autoantibodies and cholesterol crystals induce the suicidal pathway. Standard infections such as with *S. aureus* may initiate vital NETosis [23,24]. These NETs from the two pathways may have varying functional properties [25,26].

Finally, a potential third pathway of NET formation has been described with mitochondrial DNA being expressed. This may occur in response to bacterial lipopolysaccharide (LPS) which is independent of ROSs and occurs rapidly with the preservation of neutrophil function similar to vital NETosis [27]. Mitochondrial ROSs create ribonucleoprotein immune complexes with oxidized mitochondrial DNA that drives NET formation in lupus-like disease [28]. Trauma patients have NET expression induced by mitochondrial DNA [29].

Autophagy (literally the “eating of self”) is a mechanism in which cells recover nutrients by digesting their own cellular products by delivering proteins or damaged organelles to lysosomes, and is also involved in other responses such as infection. Although the mechanisms involved have not been completely defined, autophagy may trigger NET formation and protect against infection. Autophagy-driven NETosis may also damage lung tissue, leading to cell death and malignant transformation and has labelled as a double-edged sword [30].

The triggers of extracellular trap formation (discussed in more detail below) act on a variety of cellular receptors, and many triggers act on multiple receptors [31]. Microbial pathogens typically express pathogen-related molecular patterns (PAMPS) on their surfaces, which the immune system uses to distinguish these from host cells. Microbial PAMPs bind to pattern recognition receptors including Toll-like receptors, NOD-like receptors and C-type lectin receptors which activate neutrophils to express NETs [32]. Other receptors involved in extracellular trap formation include complement receptors, Fc receptors on immunoglobulin G and chemokine receptors (e.g., for interleukin 8).

In addition to neutrophils, other cell types including macrophages [33,34,35], eosinophils [36,37] and mast cells [38,39] may also produce extracellular traps. The function of these extracellular traps has not been well defined, but they are likely to have an important role in the host immune response.

Figure 1 shows NET expression, with the suicidal and vital NETosis pathways demonstrated.

In the suicidal pathway, stimuli such as PM activate the oxidative burst and the production of ROSs. This activates PAD4, which causes the chromatin decondensation of the nucleus and is also involved in the citrullination of the histones (H3Cit). NE and MPO move to the nucleus to unfold chromatin and disrupt the membrane. The processed chromatin with cytosol proteins is released into the cytoplasm and finally there is the rupture of the cell membrane with NET expression and cell death. In the vital pathway, stimuli such as those of bacterial infection activate PAD4 independent of the ROSs. PAD4 and MPO/NE have similar effects to what they do in the suicidal pathway. However, the chromatin with cytosolic proteins is released by vesicles and the cell membrane stays intact and neutrophil remains viable. NETs released by both pathways are characterised by strands of DNA with co-expressed substances (e.g., NE, MPO, H3Cit and PAD4).

## 4. Triggers of Extracellular Trap Formation

As listed above, many infectious and non-infectious causes have been identified that trigger extracellular trap formation.

### 4.1. Bacteria

Bacterial infection is prevalent in many respiratory conditions including pneumonia, chronic obstructive pulmonary disease (COPD), cystic fibrosis (CF), bronchiectasis and sinusitis. The first clearly described bacterial trigger to NET formation was *Staphylococcus aureus*, ref. [7] and NETs were demonstrated to facilitate the killing of this bacterium. Other relevant pulmonary pathogens that induce NET expression include *Haemophilus influenzae* [34,40], *Streptococcal* spp. [41], *Pseudomonas aeruginosa* [42], and *Mycobacterium tuberculosis* [33]. In clinical studies of patients with COPD and bronchiectasis, the presence of bacteria is associated with the presence of NETs, principally in sputum samples [43,44]. Antibiotic treatment reduces NET expression as measured by the eDNA in sputum samples in patients [44]. Patients with cystic fibrosis who are chronically infected with *P. aeruginosa* have higher levels of NET-related proteins in their sputum [45] and there is some evidence that the severity of CF alters the morphology of NETs in human subjects [46]. In a chinchilla model, infection with *H. influenzae* induced prominent NET expression [40]. NETs are abundant in vivo in experimental dysentery and spontaneous human appendicitis [7], and Bovine neutrophils respond to *Escherichia coli* to produce NETs in vitro [47]. MET expression is induced by exposure to *H. influenzae* [34] in vitro in human subjects. NETs have been detected in patients with M. tuberculosis and in this circumstance, the bacterium is not killed by the trap, possibly due to the protective nature of the cell wall or evasion mechanisms [48]. NETs do appear to contribute to lung damage and the NET levels in circulation could be useful in monitoring disease.

Bacteria have also developed a variety of strategies to inhibit NET function. Deoxyribonucleases (DNases) are produced both by the host and bacteria, which digest the DNA scaffold of the traps, rendering them non-functional [49,50]. DNase is produced by strains of *P. aeruginosa* to inactivate NETs. Other pathogens such as *S. aureus* also produce nucleases.

### 4.2. Viruses

Common respiratory viruses including the influenza A virus (IAV) [51], rhinovirus (RV) [52] and respiratory syncytial virus (RSV) [53] are prominent inducers of NET expression which may contribute to excessive lung inflammation and tissue damage. Toussaint et al. demonstrated that RV infection induced the release of double-stranded DNA in human samples in vitro as a marker of NET expression, and in vivo in mouse models, this infection induced NET expression [52]. There is extensive evidence in the literature documenting the effect of severe acute respiratory syndrome coronavirus 2 (SARS-CoV-2) on NET expression [54,55] and this is discussed in more detail in the subsequent text. IAV infection induces NET expression that is ineffective against secondary bacterial infection with pneumococcus both in vitro and in vivo [56]. Patients with severe IAV infections had high levels of DNA-complexes in their peripheral blood as a surrogate marker of NET expression and this was associated with poor prognosis [57].

### 4.3. Fungi

Fungal infections that trigger extracellular trap formation include Aspergillus fumigatus [58], and Candida albicans [59]. Bruns et al. demonstrated that A. fumigatus induces NETs both in vitro and in vivo in human lung tissue [58]. Another study demonstrated both in vitro and in vivo that C. albicans can induce NET expression [60]. NETs may have a role in the killing of some fungi which are too large to phagocytose, and neutrophils respond to hyphae [61]. PAD4-deficient mice (which do not make NETs) had less lung injury when infected with A. fumigatus when compared to wild-type mice [62]. Infections with A. fumigatus may also induce the expression of eosinophil extracellular traps (EETs) in addition to NETs [63]. As with bacterial infection, some fungi employ strategies to avoid NETosis including A. fumigatus which secretes hydrophobin RodA to neutralise NETs [58].

### 4.4. Non-Infectious Triggers

Evidence has demonstrated the role of NETs in response to non-microbial disease [23]. Mitogen phorbol myristate acetate (PMA) is perhaps the most potent stimulus of NET expression and was the first identified trigger [6,7].

A variety of autoimmune diseases have been associated with NETs including systemic lupus erythematosus (SLE), rheumatoid arthritis and vasculitis [64]. NET expression may trigger autoantibodies leading to autoimmunity although this connection remains contentious; whilst antibodies and antibody–antigen complexes may also trigger NET formation. Atherosclerosis [65], cancer [66] and trauma [67] are also inducers of NET expression.

Inert particles/substances have also been shown to induce extracellular trap expression such as gout crystals [68]. Cigarette smoke exposure both in vitro and in vivo triggers NET and macrophage extracellular trap (MET) expression [69]. Key triggers of extracellular trap expression are listed in Table 1.

## 5. Detection of Neutrophil Extracellular Traps

Extracellular traps are comprised of multiple strands of chromatin/DNA with co-localised mediators, principally citrullinated histones and granule proteases. They are fragile and their individual components, e.g., DNA and proteases, may arise from other inflammatory responses or cell death pathways. Therefore, the characterisation of trap responses is quite complex to demonstrate their presence [3]. The two most definitive methods to characterise the presence of extracellular traps are via the use of (1) confocal/immunostaining microscopy and (2) electron microscopy (EM).

**Figure 2:**
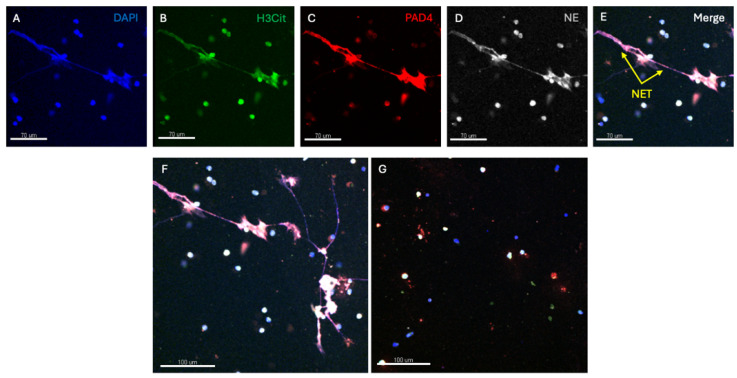
Confocal microscopy images of NET formation.

Confocal microscopy utilises fluorescent antibodies to stain for individual components of extracellular traps such as: (1) chromatin, (2) H3Cit, (3) proteases like NE and (4) PAD4 [7,17,70,71]. By the observed morphology and colocalization of multiple different components, all the essential components of NETs can be demonstrated. Cells need to be fixed and as such the individual strands of the traps are generally combined in a linear extracellular extension (rather than having a fishing net appearance). This technique requires significant infrastructure and expertise and is also very labour intensive. The interpretation of NET expression requires specific software and is prone to observer bias and background fluorescence requires appropriate controls. In addition, only relatively small numbers of cells can be studied. DNase breaks down the extracellular DNA scaffold of traps and can be useful in imaging studies to confirm their presence. Examples of confocal microscopy images are shown in Figure 2.

Figure 2 shows confocal images of NET formation induced by cigarette smoke exposure in human neutrophils. Panel A shows staining for DNA (DAPI) with extracellular extension, B staining for H3Cit, C staining for PAD 4, D staining for NE and E, the merged image demonstrating the co-localisation of H3Cit, PAD4 and NE in extracellular DNA. Panel F shows a lower-powdered image with NET expression and G, the effect of DNase in abolishing NET expression.

Transmission EM demonstrates the morphology of extracellular traps in detail with the specific staining of antigens defining their individual components [60,72,73]. This method is highly specialised and is only suitable for assessing very small numbers of cells. It can be used to confirm the expression of NETs in response to specific stimuli.

Non-cell-permeable DNA dyes like SYTOX can be used to stain cells using straightforward methods and this can demonstrate NETs clearly to experienced microscopists [16]. This method can also be used in live cells as well. It is not as specific as confocal microscopy or EM and should ideally be validated with these more definitive methods.

Flow cytometry uses fluorescent dyes to screen large numbers of cells to study biologic responses [74,75,76]. Methods have been developed to study NET expression using this methodology. Typically, published studies have been using cell-impermeable dyes like SYTOX to detect extracellular DNA/chromatin, often with antibodies for other components. This is a promising method but the flow cytometer does tend to break down NETs, so the results may not be definitive.

Various methods have been developed to define the presence of components of NETs as a marker of their presence. DNA complexes, e.g., DNA/MPO or DNA/NE may be detected in lung or peripheral blood samples as indirect markers of the presence of NETs [77,78]. Free extracellular DNA (eDNA) has also been used to define extracellular trap expression [78]; however, this method is very non-specific as the DNA may be arising from other pathways of cell death independent from NETosis.

The specimen used to assess the presence of NETs is also important. Cellular suspensions (e.g., peripheral blood or bronchoalveolar lavage) provide the best opportunities to define the presence of extracellular traps. Sputum is challenging due to its marked variability and generally degraded components. NETs can be assessed in organ tissue, but this is technically difficult due to issues with staining the extracellular traps and the need to focus into the tissue (Z-stack) [79].

## 6. Specific Respiratory Conditions

Extracellular traps have been described in an extensive variety of respiratory conditions. The role of NETs in the pathogenesis of these conditions is generally associative as there are limited published animal/in vivo models and no clinical trials. The conditions in which NETs have been best described are those with prominent neutrophil-driven inflammation.

### 6.1. Cystic Fibrosis

Cystic fibrosis (CF) arises from the absence/deficiency of the cystic fibrosis transmembrane conductance regular (CFTR) gene and its primary manifestation is progressive lung disease driven by infection/inflammation resulting in respiratory failure [80]. Inflammation in CF is driven by chronic infection with marked neutrophilic inflammation. Most of the microbial pathogens involved have been demonstrated to induce NET expression. The expression of proteases such as NE have been shown to have a primary role in the development of lung disease. Studies have demonstrated NET and NET components to be present in the sputum of CF subjects, particularly eDNA and NE [73,81,82]. Bronchoscopy studies have demonstrated the expression of extracellular traps in BAL, with associations with airway inflammation, lung function and microbial pathogens [83,84].

Neutrophils in CF may demonstrate different behaviour with possibly decreased apoptosis and longer lifespans resulting in more prominent NET expression [81,85]; this may be potentially improved by CFTR modulation [81]. CF animal models of NE knockouts have demonstrated reduced airway mucus production and neutrophilia with improved lung function, emphasising the potential benefits of targeting NE expression [86].

### 6.2. Chronic Obstructive Pulmonary Disease

COPD is a heterogeneous condition typically characterised by a combination of chronic bronchitis and emphysema. There are a variety of causes of COPD, of which the most important is cigarette smoking, which drive chronic lung inflammation and cause progressive airflow obstruction [87]. Protease imbalance with the relative overexpression of proteases such as NE and matrix metalloproteinases (MMPs) has a key role in the development of emphysema [87]; the best-known example of this is alpha-1 antitrypsin deficiency (AAT) [88]. Neutrophils are most prominent in exacerbations of chronic bronchitis. Cigarette smoke exposure induces the expression of both NETs and METs which express matrix metalloproteinase (MMP) 9 [69]. MMP9 has been previously shown to be essential for the development of emphysema [89]. Studies of sputum samples in patients with COPD have described the presence of NETs with associations with exacerbations and disease severity [43,90]. NET components including eDNA, NE, MPO and PAD4 are elevated in neutrophilic COPD [91].

### 6.3. Bronchiectasis

Bronchiectasis is characteristic by the permanent and abnormal widening of the lung bronchi arising from chronic airway infection [92]. Bronchiectasis may arise in the context of CF or in subjects without CF (non-CF bronchiectasis). Neutrophilic inflammation has a prominent role in the pathogenesis of non-CF bronchiectasis [92,93,94,95]. Neutrophils contribute to lung damage, impaired mucociliary clearance and deterioration in lung function. The presence of NE, MMP and MPO correlate with inflammation in bronchiectasis [93,96]. Levels of interleukin (IL) 6 and 8 are elevated in bronchiectasis and are associated with neutrophil levels. Sputum NET levels in patients with bronchiectasis are associated with exacerbations, worse lung function and quality of life and mortality; higher NET levels were also associated with the presence of the bacteria *H. influenzae* and *P. aeruginosa* [44].

### 6.4. Asthma

The two key features of asthma are (1) a history of variable respiratory symptoms such as cough and shortness of breath and (2) variable expiratory airflow limitation [97]. It is usually characterised by chronic airway inflammation most commonly arising from allergens such as house dust mites and pollens. Asthma has different phenotypes, of which the most well-known is eosinophilic. A proportion of patients have neutrophilic asthma which is resistant to therapy with corticosteroids and is characterised by increased inflammation and bacterial load.

A study assessed the role of NETs in patients with severe asthma principally by the assessment of sputum samples. The presence of high levels of eDNA correlated with the expression of DNA-NE and DNA-H3cit complexes as markers of the presence of NETs. The high eDNA group of patients had worse asthma control scores, more sputum and a higher use of oral corticosteroids when compared to the low eDNA group [98]. The high eDNA group also had higher levels of inflammatory cytokines/mediators including CXCL-8 and IL-1β. In a related study, NETs have been shown to contribute to asthma by activating epithelial cells to secrete chemokines including CXCL-1 and 8 [99].

Infection may also be involved in neutrophilic exacerbations of asthma. Toussaint et al. investigated the association between RV infection and NETs in asthma [52]. Subjects with asthma and controls were infected with RV and nasal samples were taken over a 2-week period. The presence of double-stranded DNA (dsDNA) was used to mark the presence of NETs, and concentrations correlated with the severity of symptoms and inflammation. They correlated these findings with in vivo mouse studies demonstrating that RV infection induced dsDNA and NET expression. The role of EETs in asthma has not been well-defined although is likely to be important.

### 6.5. Pneumonia and Acute Respiratory Distress Syndrome (ARDS)

Pneumonia is defined by the presence of infection with subsequent inflammation in the alveoli or the interstitium of the lung, and most commonly originates from bronchial infection. Pneumonia, and frequently ARDS, arise in the context of lung infection, but the host immune response may contribute significantly to pathogenesis and clinical disease. NETs and other extracellular traps are important in protecting the lungs during parenchymal infection, particularly in the early stages of the disease [24,100]. Patients with chronic granulomatous disease with a deficient/absent oxidative burst are unable to make NETs and suffer from frequent and severe respiratory infections and pneumonia [16,101]. The inhibition of NETs in mouse models of bacterial infection was associated with sepsis and increased mortality [102,103,104].

However, excessive NET activation after the initial immune response may contribute to lung damage with increased inflammation, tissue injury and thrombosis [105,106,107]. The inhibition of NET responses may reduce inflammation and levels of thrombosis and improve outcomes in animal models of lung infection. In critically ill patients with pneumonia or ARDS, higher levels of NETs are associated with worse outcomes and organ dysfunction [108,109]. Sivelestat, a neutrophil elastase inhibitor that reduces elastase activation and inhibits neutrophil aggregation in patients with ARDS/sepsis, improves lung function [110].

The optimal immune response in pneumonia/ARDs may be a strong early NET response to clear infection, but persistent and aggressive NET expression may be associated with worse outcomes [24].

### 6.6. COVID-19

SARS-CoV-2 initially infects the upper respiratory tract and usually causes mild disease. In a proportion of patients (particularly in those who are elderly), it causes lower respiratory tract infection with significant morbidity and mortality. The COVID-19 pandemic has been exhaustively investigated with many studies describing the role of NETs in lung disease [54,111,112]. Severe SARS-CoV-2 infection is associated with neutrophilic inflammation and NETosis, and post-mortem studies have demonstrated prominent NET formation and associated thrombosis [113,114,115,116]. NET-associated microvascular thrombi in the heart, lung and kidney have been described in COVID-19 patients [117]. SARS-CoV-2 directly induces NET expression [55]. Histones may enhance SARS-CoV-2 infection [118]. The NETs may a key role in inducing thrombosis. Inhibition of NETs by DNase given through inhalation was shown to improve the clinical status of a small number of patients [119,120].

### 6.7. Chronic Rhinosinusitis (CRS)

CRS is an inflammatory condition involving the paranasal sinuses and linings of the nasal passages that lasts 12 weeks or longer [121]. It is characterised by chronic inflammation that may arise from both allergic and infectious causes. It is a heterogeneous condition, and eosinophilic or neutrophilic inflammation may be present [122]. Neutrophilic inflammation is characterised by corticosteroid resistance and may be more prevalent in older patients. NET expression has been shown in patients with neutrophilic CRS and this is correlated with the expression of inflammatory mediators (NE, MPO) and antimicrobial proteins such as LL-37 [123,124,125]. The presence of EETs is closely correlated with the severity of clinical disease in subjects with a predominant eosinophil subtype [126].

### 6.8. Lung Cancer

Lung cancer is the most common cause of cancer death worldwide and most subjects have a significant cigarette smoking history. Recently, NETs have been demonstrated to be involved in a variety of cancers [127]. This appears to be primarily mediated by the inflammation induced by NETs with effects on cell proliferation, differentiation, and metastasis [128,129,130,131]. Proteases present in NETs, such as NE and MMP, remodel lung tissue, promoting proliferation and cancer spread. NETs may shield tumour cells from the clearing immune response [130]. NETs promote thrombosis and platelet activation contributing to tumour progression [132]. There is also an interaction between NETosis and autophagy [133]. Whilst the published literature focuses overwhelmingly on the pro-tumorigenic effect of NETs, studies have also demonstrated that extracellular traps are capable of directly killing cancer cells [134,135].

Emerging evidence highlights the role of extracellular traps in lung cancer using animal models. In mice, chronic smoke exposure or bacterial lipopolysaccharide (LPS) induces NET formation that activates cancer cells and facilitates metastasis [129]. Suppressing NET formation may reduce carcinogenesis [136].

### 6.9. Interstitial Lung Disease/Pulmonary Fibrosis

Interstitial lung disease (ILD) encompasses a large variety of different pathologies which may result in the scarring or fibrosis of the lung. Causes of ILD include airborne allergens such as hay (hypersensitivity pneumonitis), industrial exposure (e.g., silica and asbestos), connective tissue disease (e.g., rheumatoid arthritis), immune disorders (e.g., silicosis) and idiopathic disease [137]. Activated lung fibroblasts have a key role in this process. There is growing evidence demonstrating that NETs may directly activate fibroblasts and as well, and the inflammation from NETs damages the lung tissue leading to the secondary effects of fibrosis [138]. Most published studies have been done in animal models and the evidence in human disease is relatively low.

Extracellular trap components such as MPO and histones can directly activate fibroblasts. NETs may convert fibroblasts into myofibroblasts in vitro and in lupus models of inflammation [139,140,141]. PAD4 knockout mice (which do not make NETs) have fewer active fibroblasts and ILD [142]. NETs, and particularly proteases and histones, directly damage the lung epithelium releasing pro-inflammatory and pro-fibrotic factors, including IL-1, IL-6, IL-8 and TGF-β causing chronic inflammation resulting in ILD [143,144]. Blocking NET formation in mice reduced lung inflammation and fibrosis [145]. Extracellular trap formation may also induce autophagy that contribute to fibrosis [141].

## 7. Treatment

The effect of NETs can be reduced by a variety of pharmaceutical compounds; this effect needs to be balanced against the potential to inhibit the effectiveness of the host response to infection. Studies have described the effects of specific inhibitors of extracellular trap expression and their mediators and more general immunosuppressives such as those that reduce leukocyte chemotaxis. Key therapies that specifically inhibit extracellular trap expression/mediators are listed in Table 2.

### 7.1. DNase

Deoxyribonuclease has 3 forms [146], of which DNase 1, in the form of dornase alfa (or Pulmozyme^TM^), has been used as an inhalational medication to treat patients with CF for more than 20 years [147]. It is used to improve sputum clearance by breaking down bacterial DNA. In this context, it has been shown to be safe and well-tolerated. It also breaks down the DNA backbone of extracellular traps, which has been previously extensively documented. In vivo animal studies in a variety of models have demonstrated the benefit of DNase with less inflammation and decreased mortality [108,148,149,150,151]. Preliminary data have shown the benefit of DNase in patients with severe COVID-19 and ARDS.

As DNase inhibits a host defence mechanism against infection, there are potential issues with increasing infection risk or tissue damage. A study of the maintenance use of DNase 1 in patients with bronchiectasis demonstrated worse outcomes [152]. The DNase treatment of CF sputum has been previously shown to increase proteolytic activity [153]. DNase 1 breaks down NETs in vitro and also increases the NE activity consistent with the release of extra NE from traps [83].

### 7.2. Protease Inhibitors

Proteases such as NE present in NETs have significant potential to cause lung pathology and are a target for anti-protease agents. The most important anti-protease is AAT which primarily acts on NE but also has broad anti-inflammatory properties [88]. AAT isolated from human plasma has been used for many years to treat patients with congenital deficiencies and emphysema, which has been well tolerated but its clinical effectiveness is uncertain [88,154,155]. There is a minimal amount of published data on the use of AAT as an acute anti-inflammatory, but one study in children with CF demonstrated in vitro that it reduced NET/NE activity [83].

Studies have assessed the role of other NE inhibitors. In a mouse model of COPD, GW311616A reduced NET expression and improved lung function [156]. A series of clinical trials of NE inhibitors in CF, COPD and bronchiectasis have failed to show any benefit [157,158,159,160].

Dipeptidyl peptidase 1 (DDP1) or cathepsin C is a lysosomal cysteine protease that is involved in the activation of serine proteases including NE. The clinical relevance derives from Papillon-Lefevre syndrome in which there is a genetic deficiency of DDP1 in which patients have keratosis and periodontitis and have defective NET expression. An in vitro study of DDP1 inhibition demonstrated decreased NET formation [161]. The DDP1 inhibitor brensocatib demonstrated a reduction in blood NE activity and decreased exacerbations in a Phase 2 clinical trial [162].

### 7.3. Inhibitors of Leukotriene B4 and CXCR2

Chemotactic factors like leukotriene B4 (LTB4) and CXCR2 have a key role in attracting neutrophils to sites of inflammation. One way to reduce NET activity is to inhibit chemokines with less neutrophil migration.

An in vivo study of LTB4 inhibition demonstrated reduced neutrophil numbers but increased amounts of *P. aeruginosa* bacteraemia and inflammation in mouse lungs. A preliminary study of patients with COPD and bronchitis demonstrated some mild reductions in inflammation following treatment with an oral LTB4 inhibitor [163]. A large study of LTB4 inhibition in a CF population found a high incidence of side effects, particularly increased infections, and exacerbations [164].

The chemokine receptor CXCR2 (which binds IL-8, the most important chemokine involved in neutrophil chemotaxis) is also involved in NET formation. In vitro studies of a CXCR2 antagonist have demonstrated the reduction of NET formation in blood and sputum [165,166]. Reparixin, as an inhibitor of CXCR1/2, reduces lung neutrophils in vivo after LPS exposure [167], and NET formation in the blood of COVID-19 patients [168]. Clinical studies of CXCR2 inhibitors have demonstrated less neutrophil infiltration, but no obvious clinical benefits, and some patients have significant side effects [166,169,170].

### 7.4. Macrolides

Although macrolides are primarily an antibiotic, for many years, they have been known to have immunomodulatory properties. In vitro studies have demonstrated that macrolides decrease the neutrophil oxidative burst and lower the BAL levels of NET components such as NE and MPO [171,172]. Therapy in patients infected with *P. aeruginosa* reduced NET levels [44], and in another clinical trial, the levels of NETs were reduced in sputum following 12 months of azithromycin therapy [173]. An issue with these trials is it is very hard to determine how much of the observed effect was directly due to the antibiotic action of the macrolides.

### 7.5. Other Therapies

As discussed above, the oxidative burst with ROS production has a primary role in the formation of NET expression. In vitro and in vivo studies of NADPH inhibitors have demonstrated decreased NET formation [174,175]. N acetyl cysteine (NAC) is an antioxidant and does have an effect in reducing NET and EET expression [176]. Clinical trials have shown modest benefits in asthma, COPD, and bronchiectasis [176,177,178].

Metformin is a first-line therapy for type 2 diabetes. Recently, it also been used to treat other conditions such as cardiac disease. Metformin inhibits inflammatory signalling pathways, particularly the mTOR pathway, and consequently may reduce neutrophil numbers [179,180]. An in vivo study has demonstrated that it reduces numbers of ROSs and NET expression [180]. There is some preliminary clinical data demonstrating benefit in COVID-19 patients [181].

As discussed above, PAD4 has a primary role in the expression of NETs by the citrullination of histones, and PAD4 knockout mice do not make traps. Specific PAD4 inhibitors reduce NET and EET expression [182,183,184].

Hydroxychloroquine inhibits lysosomal proteases and reduces proteolysis and has a consequent mild immunosuppressive action with decreased chemotaxis, phagocytosis, and ROS production [185]. It is a commonly used drug in the treatment of SLE and malaria. It reduces NET formation in vivo. It has also been shown to reduce NET expression in patients with COVID-19 and SLE [186,187].

Other compounds including those derived from green tea [188] and ginger [189] have been demonstrated to reduce ROS production and NET expression.

### 7.6. Combined and Individually Tailored Therapeutics

As highlighted above, the expression of extracellular traps is a complex process with significant variability between different conditions. For optimal outcomes, the therapeutic approach needs to be tailored to each patient as it occurs with other complex diseases including autoimmune disorders like rheumatoid arthritis and cancer.

An important consideration in the therapeutic approach is whether the patient has an active infection, particularly one that is bacterial. The inhibition of extracellular trap formation in such patients has a high chance of making infection worse. Therefore, in such patients, treatment with antibiotics or other anti-microbial agents may be required in conjunction with NET inhibitors; in a later-stage/resolving infection, this may be less important.

In patients who have a non-infectious cause for extracellular trap expression such as cigarette exposure or autoimmune/interstitial lung disease, a more aggressive suppressive strategy may be appropriate. In these subjects, anti-NET therapies could be combined with other immunosuppressives to maximize the anti-inflammatory effect.

## 8. Conclusions

The study of NETs and lung disease is a rapidly expanding field. The role of other leukocyte extracellular traps such as those made by macrophages and eosinophils has had a much lower profile, but they may also have an important role in lung disease. There are a variety of techniques used to detect the presence of NETs; definitive methods such as EM and confocal microscopy require significant expertise and can only screen small numbers of cells, whilst other methods with the detection of individual components of extracellular traps like eDNA and NE are very non-specific. The development of more specific and less technically challenging testing would be a significant advance. The roles of extracellular traps have been best described in acute lung injury and airway diseases; parenchymal/interstitial lung disease and cancer are emerging areas of interest. The targeting of extracellular traps may be a new frontier in anti-inflammatory therapy for respiratory diseases, particularly for neutrophil-driven inflammation. However, the current clinical trials/evidence is at a very early stage.

## Figures and Tables

**Table 1:** Key triggers of extracellular trap formation.

Infectious	Non-Infectious
Bacterial *S. aureus*, *H. influenzae*, *Strep* spp., *P. aeruginosa*, *M. tuberculosis* Viral Influenza, RV, RSV, SARS-CoV-2 Fungal *A. Fumigatus*, *C. Albicans*	Chemical Mitogens like PMA Autoimmunity/antibodies SLE, rheumatoid arthritis, vasculitis Cancer Other Gout crystals, cigarette smoke

**Table 2:** Key potential therapies for extracellular trap driven disease.

Target	Therapy	Effect
DNA	DNase	Improves sputum clearance in CF Decreases inflammation and improves mortality in animal studies Improved clinical outcomes in preliminary studies in COVID-19 As a maintenance therapy for bronchiectasis, shows worse clinical outcomes
Neutrophil elastase	AAT	Used for many years for patients with congenital deficiency and emphysema with modest effect Reduces NE activity in vitro in NETs
	Other direct NE inhibitors (GW311616A)	In COPD animal models, reduces NET expression and improves lung function No benefit in clinical trials in CF, COPF and bronchiectasis
	DDP1 inhibition	In an in vitro study, decreased NET expression In a Phase 2 clinical trial, reduces blood NE activity and decreases exacerbations
Oxidative burst/ROS	NAC	Reduces ROS production in vitro Reduces NET and EET expression in vitro Mild clinical benefits
PAD 4	PAD4 knockout mice	No NET expression, decreased inflammation
	PAD4 inhibitors	Reduce NET expression in vitro
